# Laser‐Induced Graphene‐Based Resistive Leaf Wetness Sensors for Pesticide Spray Deposition

**DOI:** 10.1002/gch2.70165

**Published:** 2026-10-15

**Authors:** Nathan M. Jared, Kristi K. Bez, Roham G. Goudarzi, Griffin Ellis, Krishangee Bez, Emily A. Smith, Jonathan C. Claussen

**Affiliations:** ^1^ Department of Mechanical Engineering Iowa State University Ames Iowa USA; ^2^ Department of Chemistry Iowa State University Ames Iowa USA

**Keywords:** interdigitated electrode, laser‐induced graphene, leaf wetness, pesticides, resistive sensor

## Abstract

Monitoring pesticide spray deposition across crop canopies requires low‐cost sensors capable of quantifying deposition through leaf wetness, yet carbon‐based resistive leaf wetness sensors (LWS) have not been benchmarked against commercial systems. Here, we demonstrate a scalable resistive laser‐induced graphene (LIG) LWS patterned as an interdigitated electrode for pesticide spray monitoring. The sensor achieved normalized wetness values of 0.15, 1.79, and 2.66% cm^−2^ after 1, 2, and 5 s of spraying, compared with 0.18, 1.26, and 1.91% cm^−2^ for a commercial Onset LWS, indicating greater sensitivity at longer exposures. Calibration followed a formulation‐dependent power‐law relationship between resistance and deposited volume. Across three independently fabricated sensors, the selected double‐lased 0.7 mm IDE produced a reproducible tap‐water calibration, with *A* = 45.167 ± 1.841, *n* = −0.957 ± 0.021, and *R* ^2^ = 0.9921. Short‐term stability depended on solution and environment, with tap‐water calibration changing only 1.5% under greenhouse conditions and 6.3% outdoors after three days, while pesticide formulations exhibited greater drift. Repeated pesticide exposure also caused formulation‐dependent drift, indicating that formulation‐specific calibration and deployment protocols are required. Overall, the low‐cost, scalable LIG LWS offers a promising platform for distributed pesticide spray monitoring and precision agriculture.

## Graphical abstract

A laser‐induced graphene leaf wetness sensor is fabricated by CO_2_ laser‐patterning interdigitated electrodes on flexible polyimide and adding silver‐paste contacts through a shadow mask. Calibration with multiple pesticide formulations and tap water shows decreasing resistance with increasing deposited volume, while spray tests demonstrate close agreement between sensor‐estimated and measured pesticide volumes across increasing spray durations.

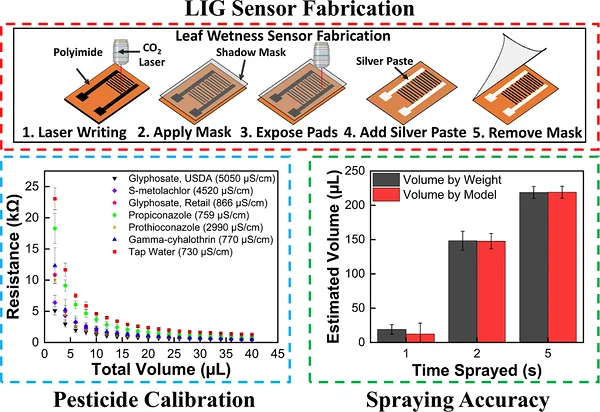

## 1 Introduction

In modern agriculture, pesticides are heavily relied on to optimize crop yield while preventing weed, pest/insect, and fungal infestations during rigorous farming practices [1]. It is predicted that without the application of pesticides, there would be immediate losses in fruit, vegetable, and cereal production by 78%, 54%, and 31%, respectively [2]. However, when applied in excessive quantities, pesticides can become environmental contaminants, polluting soil and water resources and disrupting the availability of essential crop nutrients [3]. In fact, approximately 64% of global agricultural land is at risk of pesticide pollution due to overapplication of agrochemicals [4]. A reduction in pesticide application is not a straightforward solution, however, as this can result in pesticide resistance in weeds, insects, or fungi being treated, leading to a reduction in crop yield [5]. Optimizing current spraying techniques is also difficult. For instance, ground‐based techniques rely heavily on the performance of sprayer nozzles that influence spray structure, droplet size, and droplet velocity [6]. Additionally, unmanned aerial vehicle (UAV) sprayers create downwash flow that potentially disturbs the crops within the canopy, causing swinging that affects the position and behavior of droplets deposited within the canopy [6]. Therefore, there is an urgent need to properly measure pesticide spray directly on crop leaves and map spray distribution of pesticides to reduce overapplication and improve spray uniformity. This can be accomplished through measuring the wetness of crop leaves after pesticides are sprayed and strategically placing sensors at different levels of a crop canopy to quantify pesticide spray so future spraying applications can be adjusted accordingly [7].

There have been numerous commercial and research‐based LWS developed for irrigation management, environmental monitoring, and disease prevention; however, existing systems are designed for leaf wetness duration (LWD) rather than spatially distributed pesticide spray deposition on crop leaves [8, 9, 10]. Previous studies have also demonstrated that wetness distribution varies significantly throughout plant canopies, highlighting the need for distributed sensing approaches capable of characterizing localized deposition behavior [11, 12, 13]. While electrical wetness sensing approaches have been explored for agricultural monitoring applications, there remains a technological gap in the development of a scalable, low‐cost, flexible resistive LWS specifically designed and experimentally validated for quantifying pesticide spray distribution under realistic field conditions.

Measuring the wetness on the surface of leaves is instrumental to monitor several agricultural parameters such as plant disease management [14], irrigation distribution [15], and pesticide application [16]. Only 20–30% of applied pesticides come into contact with the leaves of plants, where droplets will either adhere to the leaves or roll off into the surrounding environment [17, 18]. With only a fraction of pesticides captured by the surface of leaves influencing overall crop protection, there is a desire to retain droplets on the surface to improve pesticide effectiveness [19]. However, pesticides are often overapplied to fully saturate crop leaves and maximize pesticide effectiveness. The overall pesticide wetness of leaves allows for high droplet retention, but increases the risk of soil pollution, groundwater pollution, and a threat to human health due to more droplets rolling off leaf surfaces as the leaves become saturated [19, 20, 21, 22]. Therefore, advances in technology and sensors are needed to properly monitor the wetness of pesticide spray.

Common approaches for evaluating pesticide spray deposition and environmental contaminants include chemically selective analytical platforms, water‐sensitive paper (WSP), capacitive sensors, and resistive sensors. For example, Ozório et al. (2025) developed ZnO nanorod/Ag nanoparticle‐functionalized paper substrates for sensitive surface‐enhanced Raman spectroscopy detection of environmental contaminants, including pesticide residues [23]. Laser‐induced graphene (LIG) has also been adapted as a conductive platform for selective molecular sensing. Marques et al. (2020) developed a dual electrode LIG platform functionalized with molecularly imprinted polymers for the recognition of target molecules [24]. In that system, the molecularly imprinted polymers provided molecular selectivity, while the LIG served as the conductive transducer. These chemically selective platforms provide information about contaminant identity, whereas the unfunctionalized LIG LWS developed in the present study quantifies localized spray deposition through resistance changes caused when liquid droplets bridge the IDE fingers. For spray‐deposition measurements, Turner et al. (1970) created a method of assessing aqueous spray droplets on glazed photographic paper, which was later commercialized as WSP by the chemical company Ciba‐Geigy in 1985 [25]. When sprayed, WSPs turn dark blue where aqueous droplets would contact the surface of the paper. WSPs would be placed within a crop field, and pesticides would be applied throughout the crop canopy. After spraying, the WSPs would be removed, and several factors such as coverage area, spraying distribution, and droplet diameter could be calculated [26]. However, WSPs are limited by the diameter of droplets in contact with the surface of the paper, and overlapping droplets make it difficult to provide an accurate estimation of droplet distribution while being time‐consuming and labor‐intensive to install [27]. To resolve this, capacitive and resistive‐based leaf wetness sensors (LWS) were created. Capacitive LWS, like those from Campbell Scientific [28], measure the dielectric constant of a material between two electrodes when droplets are present on the surface [29]. These types of sensors use an interdigitated electrode (IDE) design coated with a dielectric material, which allows for greater sensor sensitivity but is limited in size due to their cost [10, 30]. Resistive LWS, like that from Davis Instruments [31], also use an IDE design but are covered by a material that creates a thin film as droplets encounter the sensor surface, bridging the electrode grid and reducing the electrical resistance between two electrodes [10, 32, 33]. These sensors, although exhibiting reduced sensitivity compared to capacitive alternatives, are cost‐effective, have simple circuit designs, and the geometry of the IDE design can be optimized for a specific application. Critically, resistive LWS are also simpler to fabricate at scale than their capacitive counterparts. Capacitive sensors require an additional dielectric coating to be deposited over the IDE to support the change in dielectric constant that is sensed, adding a fabrication step that increases process complexity and cost. Resistive sensors, by contrast, operate directly on the bridging of the bare electrode grid by accumulated droplets and require no such dielectric layer, making them inherently better suited to low‐cost, high‐throughput manufacturing of large numbers of distributed sensors. Several resistive LWS have previously been reported for agricultural applications, including disease forecasting, irrigation management, and environmental wetness monitoring [10, 34, 35]. However, most existing resistive sensors have been designed to measure leaf wetness duration and humidity rather than quantify pesticide spray deposition and distribution throughout a crop canopy [36]. Furthermore, many reported resistive sensors rely on rigid metallic electrodes, printed circuit board fabrication, or commercial sensing platforms that increase manufacturing costs and limit the number of sensors that can be deployed within a field, such as those from Davis Instruments [31]. However, there remains a significant gap in the development of scalable, flexible, and inexpensive resistive sensing platforms capable of monitoring pesticide spray distribution under realistic agricultural field conditions.

LIG has emerged as a promising material for developing wetness sensors due to its porous nature/wettability tuning, excellent conductivity, flexibility, cost‐effectiveness, and high‐throughput manufacturing [37, 38, 39, 40, 41]. LIG is synthesized using a CO_2_ laser that converts sp^3^ carbon atoms into sp^2^ carbon atoms by localizing a high temperature above 1000°C onto a carbon‐rich substrate [38, 42, 43]. Unlike traditional graphene synthesis techniques such as chemical vapor deposition [44, 45, 46], microwave synthesis [47, 48], and mechanical exfoliation [49, 50, 51], the manufacturing process of LIG is a one‐step process allowing for significant advantages in manufacturing cost and efficiency. One key advantage of LIG manufacturing is the ability to tune the material toward hydrophilic or hydrophobic behavior to match that of surfaces of natural crop leaves. Previously from our group, we have developed laser parameters to create both hydrophilic LIG, like that of pesticide spray collection patches [37], and hydrophobic LIG, like that to create open microfluidic tracks [52]. Additionally, LIG on carbon‐rich substrates such as polyimide allows for patterning LIG on flexible substrates in configurations designed to mimic the structure of natural leaves for a more realistic sensing environment [53]. As such, LIG fabrication offers a cost‐effective, high‐throughput manufacturing process to create a LIG LWS that is porous and conductive, with potential for deployment in crop fields. To date, however, LIG‐based leaf wetness sensing has been demonstrated almost exclusively in a capacitive configuration, such as the parylene‐C‐coated capacitive LIG LWS reported by Huang et al. [52]. Recent work has also demonstrated paper‐based LIG humidity sensors for low‐cost and scalable monitoring of atmospheric moisture [54]. However, such devices detect changes in relative humidity rather than the accumulation of liquid spray droplets. The present work instead uses an uncoated resistive LIG IDE to quantify deposited pesticide spray through solution‐dependent resistance–volume calibrations. A resistive LIG LWS, which would pair the single‐step, coating‐free scalability of LIG with the simpler readout of a resistive platform, has not yet been demonstrated for quantifying pesticide spray deposition, representing the gap this work addresses.

Herein, we introduce the concept of creating a LIG LWS that uses an IDE design to quantify pesticide spray based on a change in measured electrical resistance as droplets come into contact on the surface of the IDE grid. Creating LIG bypasses the need to print graphene inks using methods that require energy‐intensive post‐print annealing such as using high‐temperature ovens, toxic solvents such as cyclohexane, or printing inks using costly equipment such as gravure or inkjet printers [55, 56, 57, 58]. The LIG LWS is created with an IDE design using CorelDRAW, and the wettability properties of the LIG can be tuned by changing the settings of the CO_2_ laser. The resulting LWS can be calibrated toward a specific solution, in our case, pesticides, in which resistance values are measured as solution volume is accumulated onto the surface of the LWS. A custom calibration equation is created for each solution, and a wetness percentage can be calculated. This low‐cost, LIG LWS could be deployed on a larger scale within a crop canopy to monitor how pesticide spray is distributed throughout and allow agricultural workers to optimize spraying techniques based on sensor performance.

## 2 Results and Discussion

### 2.1 Fabrication of LIG IDE

A simple two‐step laser process patterned the entire IDE directly on flexible polyimide, producing a leaf‐mimicking sensing surface in a single, coating‐free workflow. The IDE was fabricated from LIG synthesized from polyimide using a CO_2_ laser (Figure 1). The first lasing of LIG used laser parameters of 15% speed, 7% power, and 1200 dots per inch (DPI) with a defocus of 1 mm away from the polyimide surface. A second laser pass was done on top of the first pass with settings of 20% speed and 3% power. As demonstrated in our previous work, the first laser pass produces hydrophilic laser‐induced graphene (LIG) with near‐zero water contact angles and reduced electrical resistivity, while the second laser pass creates less hydrophilic LIG that more closely matches the wettability of natural leaves while still maintaining high conductivity [52, 59]. The structural and electrical properties of LIG are governed by parameters including laser power, speed, DPI, focal position, and number of laser passes. Previous measurements of LIG fabricated using polyimide substrates and processing conditions showed a porous LIG layer thickness of approximately 25–50 µm [52]. However, thickness does not independently determine electrical performance because laser processing also alters graphitization, defect density, pore connectivity, surface morphology, and contact resistance. In the present study, the second laser pass simultaneously modified these properties, as indicated by the SEM, XPS, and Raman results. Therefore, the improved calibration consistency of the double‐lased 0.7 mm IDE cannot be attributed solely to electrode thickness. The LIG IDE consisted of two 75 by 1.2 mm vertical electrodes (legs). Each leg had thirty‐five 24.1 by 0.3 mm horizontal fingers interlaced. At the end of each leg, a 5‐by‐5 mm LIG square was placed, where clips were placed to measure the resistance of the sensor. The gap between the fingers (pitch) was either 0.7 or 0.3 mm. Once laser‐cut, a stencil was applied on top of the LIG, and both electrode legs were cut out of the stencil. Both single‐lased and double‐lased LIGs were used to compare droplet‐calibration consistency in the following sections.

**FIGURE 1:**
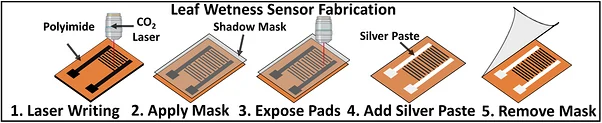
Fabrication process and final structure of the LIG IDE LWS. (1) CO_2_ laser carbonization of Kapton polyimide into the IDE pattern (finger dimensions 24.1 × 0.3 mm, 0.7 mm spacing, 2010 mm^2^ active area). Second laser passes on the LIG IDE. Single‐lased LIG (one pass): highly hydrophilic, near‐zero contact angle, reduced resistivity; double‐lased LIG (second pass): lower hydrophilicity matched to natural leaves, conductivity retained. (2) Shadow (stencil) mask applied to protect the IDE sensing region. (3) Laser cut on shadow mask to expose LIG legs and pads. (4) Silver (Ag) ink applied to exposed LIG legs and pads to lower contact resistance, improve LIG‐to‐clip conductivity, and enhance mechanical durability. (5) Removing shadow mask for final sensor completion.

Beyond reduced material cost, the LIG fabrication process offers several advantages for scalable sensor production. The IDE is generated directly from the polyimide substrate using a defined laser pattern, eliminating graphene‐ink preparation, electrode printing, material transfer, and post‐print thermal annealing. Sensor dimensions can also be modified directly in the design file without producing new photolithographic masks or printing screens. In addition, the resistive operating mechanism does not require the dielectric coating used in capacitive LWS platforms, reducing the number of fabrication steps. The resulting two‐terminal resistance output is compatible with relatively simple readout electronics and could support future multiplexing of multiple sensors throughout a crop canopy. However, manufacturing throughput, batch yield, and large‐scale production consistency were not directly evaluated in this study and therefore represent potential manufacturing advantages rather than demonstrated mass‐production performance.

### 2.2 Material Characterization of LIG IDE

Material characterization confirmed that the second laser pass preserves the conductive graphene network while tuning surface wettability, yielding a double‐lased LIG that withstands stencil‐based fabrication far better than its single‐lased counterpart. LIG characterization was conducted using scanning electron microscopy (SEM), X‐ray photoelectron spectroscopy (XPS), and Raman spectroscopy. These three techniques were used to observe how the LIG material is affected after applying and peeling off the stencil used for applying the Ag paste to the sensor. SEMs were acquired to visually observe how the LIG microstructure was affected for both single‐ and double‐lased LIG. Figure 2A shows the fiber‐like structure of single‐lased, hydrophilic LIG, while Figure 2B shows after the stencil was applied and peeled off. Figure 2C,D shows the before and after application of the stencil for double‐lased LIG. From the SEM images, there do not appear to be significant structural changes before and after mask application for the double‐lased LIG. However, substantial visual destruction of the fiber‐like structure is observed for the single‐lased LIG after the stencil is peeled off. This is likely because single‐lased LIG typically forms a highly porous, loosely interconnected fibrous graphene network with a large surface area and relatively fragile microstructures generated during the irradiation of polyimide [37]. In contrast, secondary laser exposure during double lasing partially restructures the graphene network through additional localized heating and carbon reorganization, resulting in a denser morphology with reduced porosity and improved mechanical stability [52]. Therefore, the structural differences observed after stencil removal are likely associated with the weaker mechanical integrity of the single‐lased material. Similar behavior has been reported for porous LIG materials where high surface area networks and defect‐rich graphene structures are susceptible to mechanical damage and structural deformation under external forces or repeated handling [38, 60]. In contrast, the more compact and interconnected morphology of double‐lased LIG provides improved structural robustness that reduces susceptibility to mechanical disruption during stencil application and removal [52]. Consequently, minimal visual changes were observed for the double‐lased LIG following stencil peeling. These results suggest that secondary laser processing not only modifies wettability characteristics but also enhances the mechanical durability of LIG for subsequent fabrication processes and sensor integration applications.

**FIGURE 2:**
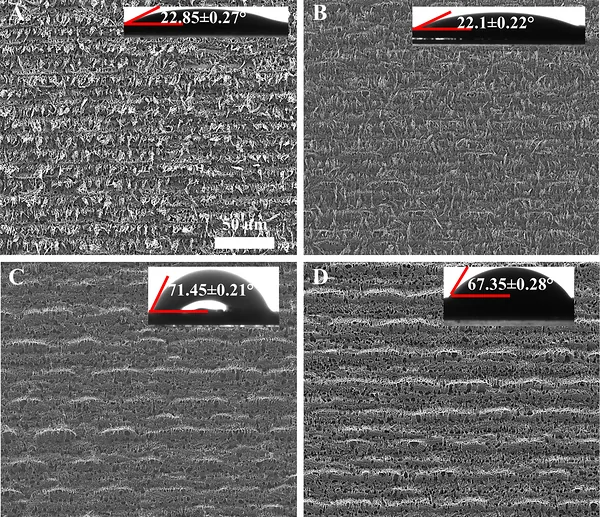
SEM surface topography of LIG on Kapton polyimide under different laser conditions, with water contact angles inset. (A, B) Single‐lased LIG that is porous, rough, and superhydrophilic; contact angles 22.85 ± 0.27° and 22.19 ± 0.22° (A, as‐fabricated; B, after mask removal – minimal morphological change). (C, D) Double‐lased LIG densifies the network, reducing porosity and smoothing ridges and creates a more hydrophobic surface with water contact angles of 71.45 ± 0.21° and 67.35 ± 0.28° (C, as‐fabricated; D, after mask removal showing negligible damage to LIG structure). All images: surface view, ∼150× magnification, 10° tilt, 200 µm scale bars. Morphological differences confirm that the second laser pass tunes surface roughness, porosity, and wettability toward natural‐leaf behavior while preserving conductive pathways for resistive sensing. The scale bar in panel A is identical across all SEM images.

X‐ray photoelectron spectroscopy (XPS) and Raman spectroscopy were utilized to characterize both single‐ and double‐lased LIG of their elemental composition and structural properties. XPS confirmed elemental composition peaks of carbon (C1s) at around 284.5 eV and oxygen (O1s) at around 532.3 eV. For both single (Figure S1A) and double (Figure S1B) laser LIG, the intensity of the C1s and O1s changes by less than 1% after the mask is peeled off the material. Consistent with our previous work, the fabrication of double‐lased LIG causes a decrease in oxygen content of the single‐lased LIG, in this case from 10.15% to 6.33%, while the carbon content increased from 89.95% to 97.06% [52]. A double‐lased LIG method essentially reduces the number of oxygen functional groups within the surface, including hydroxyl, carbonyl, and carboxyl groups, of the LIG, causing the material to repel water droplets, indicating a less hydrophilic surface surface [52, 61, 62]. However, the change in wettability cannot be attributed to surface chemistry alone. The SEM images also show that the second laser pass transformed the highly porous and fibrous single‐lased LIG network into a denser, less porous surface with smoother ridge‐like features. Surface topography can modify the apparent contact angle by influencing liquid spreading, capillary infiltration, and the extent of solid–liquid contact, while surface roughness generally amplifies the intrinsic wetting behavior established by the underlying surface chemistry [63]. Therefore, the increase in water contact angle from 22.85 ± 0.27° for single‐lased LIG to 71.45 ± 0.21° for double‐lased LIG likely results from a synergistic combination of reduced oxygen functionality and altered surface morphology. The lower concentration of polar surface groups reduces the chemical affinity of the LIG for water, while the denser and less porous morphology likely limits capillary infiltration and spreading within the graphene network. Because surface roughness was not independently quantified and the effects of surface chemistry and morphology were not experimentally isolated, their relative contributions cannot be determined from the present measurements. Nevertheless, the combined XPS, SEM, and water‐contact‐angle results indicate that both factors contribute to the observed reduction in hydrophilicity following the second laser pass. Raman spectroscopy (Figure S1C,D) characterized three bands of graphitic material of LIG. The D band (∼1300 cm^−1^) represents any defects that are present in the graphitic material [64], the G band (∼1584 cm^−1^) represents the sp^2^‐carbon atoms present in the graphitic material [65], and the 2D band (∼2590 cm^−1^) represents few‐layers graphene [66]. The intensity of the D and G peaks showed a change of 1–3 a.u. for both single and double‐lased LIG after the mask was peeled off the material. However, the 2D peak changed 62 a.u. for single‐lased LIG and 9 a.u. for double‐lased LIG. The D/G ratio going from single to double‐lased LIG showed an increase from 2.22 ± 0.09 to 2.4 ± 0.2 while the 2D/G peak decreased from 0.12 ± 0.03 to 0.09 ± 0.05. It is important to note that for both single and double‐lased LIG, the D/G intensity ratios show a similar degree of disorder within the material and the 2D/G intensity ratio illustrates the LIG contains multi‐layer graphene [46]. The Raman spectrum of LIG before and after applying the shadow mask can be found in Table S1.

### 2.3 Droplet Characterization and Calibration

The double‐lased LIG IDE with a 0.7 mm pitch produced the most consistent power‐decay calibration across all droplet sizes, with tap water yielding A = 45.167, n = ‐0.957, and R^2^ = 0.9921, outperforming both single‐lased IDE designs (Table 1). This calibration stability indicates that the double‐lased 0.7 mm design was least affected by individual droplet volume and was therefore selected for subsequent pesticide spray sensing experiments. To evaluate IDE design and laser settings to move forward with, wetness calibrations were performed using tap water droplets of 2, 4, 6, 8, 10, and 20 µL. For each design, n = 3 sensors were tested, and each droplet size was deposited 20 times onto the IDE surface. Thus, the final accumulated volume depended on droplet size, ranging from 40 for 2 µL droplets to 400 for 20 µL droplets. After each droplet was placed onto the IDE, the liquid bridged adjacent electrode fingers, increasing electrical pathways across the IDE and decreasing the measured resistance. The experimental setup for calibrating the LIG LWS with various solutions can be found in Figure S2. The relationship between total deposited volume and resistance followed a power‐decay model:

$$R=AV^{n}$$

where V is the total volume on the IDE surface, R is the measured resistance, and A and n are experimentally determined calibration parameters that depend on the tested solution. Note that there are no values for a double‐lased LIG IDE with a 0.3 mm pitch. This is due to the resistance when no fluid is on the IDE surface being below 1 kΩ indicating bridging of the fingers. This bridging is likely due to an increase of graphene layers after completing an additional laser pass and the close proximity of the horizontal fingers resulting in the drop in initial IDE resistance [67, 68].

**TABLE 1:** Experimental variables for power decay model and statistical analysis for three IDE designs.

	SL 0.7 mm Pitch	SL 0.3 mm Pitch	DL 0.7 mm Pitch
Coefficient (A)	36.399 ± 5.363	12.727 ± 5.187	45.167 ± 1.841
Exponent (n)	−0.859 ± 0.045	−0.867 ± 0.103	−0.957 ± 0.021
p‐value	2.2e‐16	2.2e‐16	2.2e‐16
R^2^	0.9826	0.9101	0.9921

Rascio et al. (2019) demonstrated a similar trend for measuring electrical resistance of water moisture on crop leaves for rapid estimation under environmental conditions [34].

Based on the three IDE designs, the double‐lase LIG with 0.7 mm pitch showed the best overlap among the calibration curves for different droplet sizes, indicating that the sensor response was primarily governed by total liquid volume rather than individual droplet size. In contrast, the single‐lased LIG IDEs with 0.7 and 0.3 mm pitches showed greater separation among droplet‐size calibrations, suggesting less repeatable wetting and bridging behavior across the IDE fingers. Figure 3A corresponds to the double‐lased 0.7 mm pitch IDE, Figure 3B corresponds to the single‐lased 0.7 mm pitch IDE, and Figure 3C corresponds to the single‐lased 0.3 mm pitch IDE. This improved repeatability is further supported by the lower standard deviations in the fitted coefficient A and exponent n for the double‐lased 0.7 mm design (Table 1). The improved performance of the double‐lased 0.7 mm IDE is likely due to both material and geometric effects. Laser‐processing parameters influence the morphology, porosity, graphitic quality, and electrical properties of laser‐scribed graphene electrodes, and optimization of these parameters has been shown to alter subsequent sensing performance. In the present study, the second laser pass simultaneously modified the morphology, wettability, and electrical behavior of the LIG, contributing to the improved calibration consistency of the double‐lased 0.7 mm IDE. Prior studies have shown that repeated laser exposure can alter LIG thickness, morphology, conductivity, graphitization, and wettability, which can directly influence sensor performance and electrical stability [52, 69]. Additionally, LIG structure, conductivity, and wettability can be tuned through laser‐processing conditions, providing a mechanism for improving liquid interaction and electrical response [37, 70]. In this study, the 0.7 mm pitch likely provided sufficient spacing to prevent unintended dry‐state bridging between adjacent IDE fingers while still allowing droplets to reliably connect the electrodes during wetting. The IDE architecture directly controls the resistive wetness‐sensing mechanism. The number and length of the interlaced fingers determine the available sensing area and number of potential liquid‐mediated conductive pathways, while the gap between adjacent fingers controls the distance that deposited droplets must spread to electrically bridge the two electrodes. Decreasing the finger gap can increase the probability that low‐volume droplets bridge adjacent fingers. However, excessively small spacing can also reduce dry‐state electrical isolation because of lateral carbonization or LIG overgrowth during fabrication, resulting in electrical shorting. This behavior was observed for the double‐lased 0.3 mm design, which exhibited a dry‐state resistance below 1 kΩ and was therefore excluded from further testing. In contrast, the 0.7 mm design provided sufficient separation to maintain dry‐state isolation while still allowing deposited droplets to form repeatable conductive pathways. This balance contributed to the lower variation in the fitted calibration parameters for the double‐lased 0.7 mm IDE.

**FIGURE 3:**
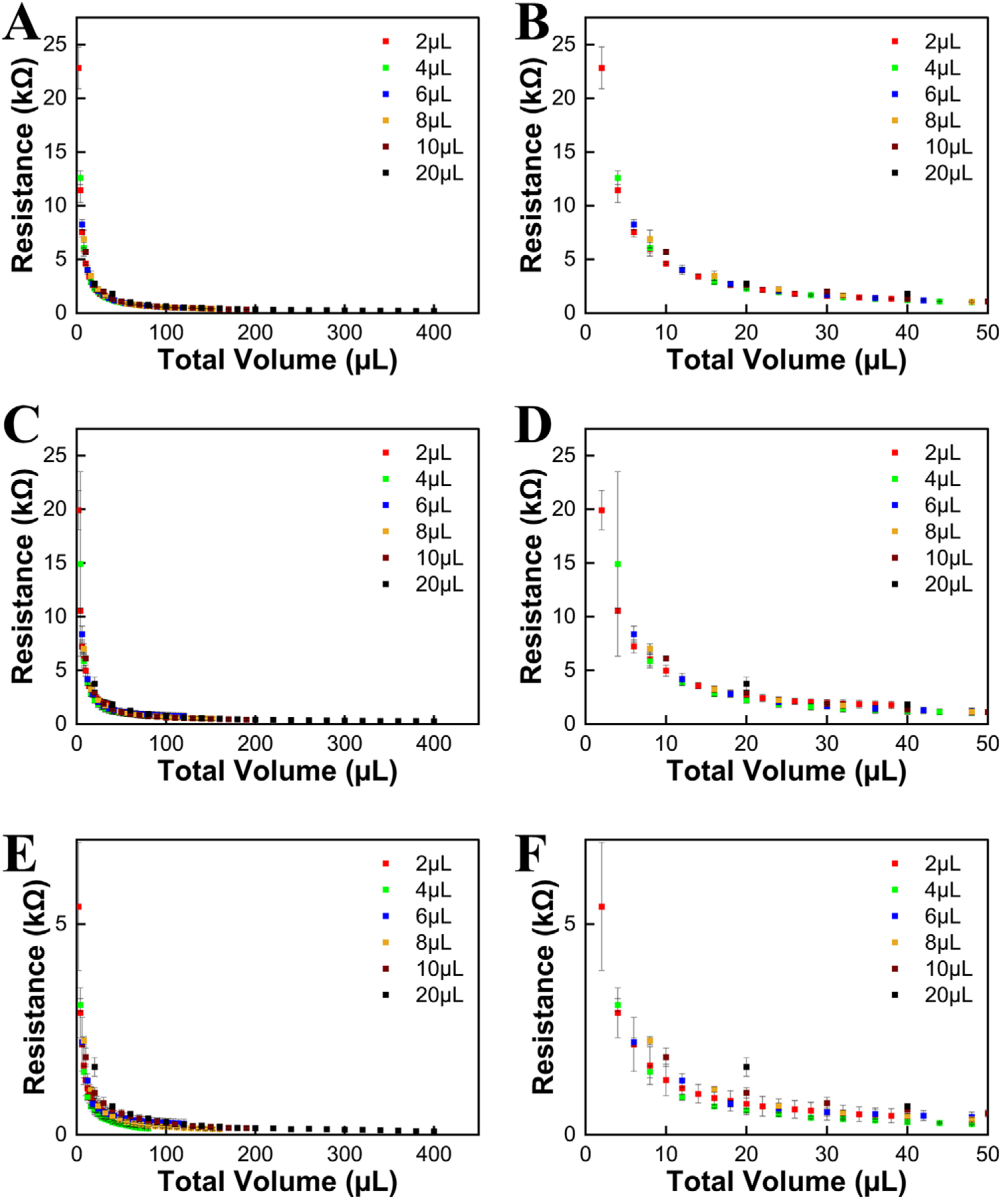
Droplet calibration curves of the LIG LWS for sequential tap water additions. (A–B) double‐lase, 0.7 mm IDE pitch; (C–D) single‐lase, 0.7 mm pitch; (E–F) single‐lase, 0.3 mm pitch. Droplets (2, 4, 6, 8, 10, 20 µL) deposited sequentially to 40–400 µL total. Panels A, C, and E, full range; B, D, and F, enlarged low‐volume region (0–50 µL) of greatest resistance change. 4263B LCR meter, controlled lab conditions, n = 3 sensors per design; error bars = standard deviation.

For all sensor configurations, resistance decreased as the accumulated liquid volume increased due to the formation of conductive liquid bridges between adjacent IDE fingers. The resulting calibration response followed a power‐law relationship (R = AV^n^), where R is the measured resistance, V is the accumulated liquid volume, and A and n are experimentally determined fitting parameters. The fitted coefficients, exponents, and corresponding R^2^ values are reported in Table 1, while the calibration coefficients and exponents of all droplet sizes can be found in Table S2. Among the tested designs, the double‐lase LIG sensor with a 0.7 mm pitch (A–B) exhibited the most consistent response across all droplet sizes, with minimal divergence between calibration curves and reduced variability between sensors. In contrast, the single‐lase designs (C–F) exhibited greater separation between droplet‐size calibrations, indicating that droplet spreading and electrode bridging were more dependent on the individual droplet volume. The improved consistency of the double‐lase design is attributed to the modified surface morphology and wettability resulting from secondary laser processing, which promotes more uniform liquid distribution and repeatable conductive pathway formation across the sensing area. The repeatability of the chosen fabrication design can be found in Figure S3 across n = 4 sensors.

After validation of the performance of the three sensors with tap water, the double‐lase LIG with 0.7 mm pitch was selected for all subsequent experiments. This is due to that sensor having the highest R^2^ value and lowest standard deviation for the experimental variables A and n. Six pesticide solutions were tested: a glyphosate‐based herbicide (USDA), an S‐metolachlor‐based herbicide, a glyphosate‐based herbicide (retail), a propiconazole‐based fungicide, a prothioconazole‐based fungicide, and a gamma‐cyhalothrin‐based insecticide. These experiments were conducted to determine how changing the solution composition, while maintaining a constant solution volume, affected the power model established in Table 1. Each pesticide was tested identically to tap water, with 2 µL droplets of each solution being applied to an IDE n = 20 times for a total of 40 µL accumulated onto the surface of the IDE. As shown in Table 2, the coefficient and exponent of the power models developed for each solution tested differed from solution to solution. It should be noted that each commercial formulation contains not only the active ingredient, but also water, surfactants, wetting agents, dispersants, dissolved salts, and other proprietary adjuvants. These additional constituents influence the solution conductivity (Table 2), which in turn affects the calibration coefficient (A), providing the rationale for reporting conductivity values alongside each formulation. This can further be illustrated by Figure 4 where the solution power model gave different results. Note that there are factors that could influence the measured resistance of the IDE. For instance, the coefficient (A) of the calibrations increased as the conductivity of the tested solution decreased, as shown in Table 2. The exponent (n) is likely affected by the concentration of the pesticides tested. Each pesticide is made of an active ingredient with additional surfactants that can alter how each pesticide spreads on the surface of leaves [71, 72], and, in the case of the LIG sensors, alter the spreading and contact angle of spray droplets. These changes affect the bridging of the IDE fingers, shift the resistance measured at the same droplet volume, and ultimately alter the calibration for each pesticide. Palleja et al. (2023) performed a similar study with IDE pesticide spray sensors where they discovered that varying chemicals within “ready‐to‐spray” pesticides can change the droplet surface area, thereby altering how droplets spread across the sensor [73]. For our study, this further illustrates that the LIG LWS needs to be calibrated for a particular pesticide solution before spraying applications, as each pesticide has a unique calibration model.

**TABLE 2:** Experimental coefficient (A) and exponent (n) of each pesticide solution calibrated with 2 µL droplets.

Solution Calibrated	Coefficient (A)	Exponent (n)	Conductivity (µS/cm)
Glyphosate‐based (USDA)	9.591 ± 2.621	−0.867 ± 0.051	5050
S‐metolachlor‐based	15.386 ± 4.656	−0.906 ± 0.082	4520
Prothioconazole‐based	16.524 ± 1.507	−0.987 ± 0.065	2990
Glyphosate‐based (retail)	18.721 ± 2.959	−0.981 ± 0.068	866
Gamma‐cyhalothrin‐based	23.662 ± 8.304	−1.045 ± 0.067	770
Propiconazole‐based	40.125 ± 4.029	−1.068 ± 0.054	759
Tap Water	45.167 ± 1.841	−0.957 ± 0.021	730

**FIGURE 4:**
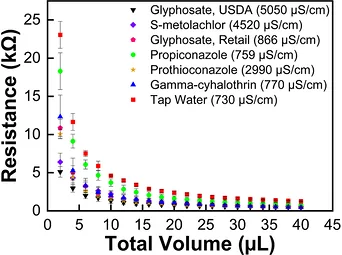
Resistance response of the LIG LWS to commercial agricultural spray formulations (glyphosate‐based from USDA and retail sources, S‐metolachlor‐, propiconazole‐, prothioconazole‐, and gamma‐cyhalothrin‐based products, and tap water; labeled by active ingredient). Resistance (kΩ, *y*‐axis) vs. total deposited volume (µL, *x*‐axis) from sequential droplet additions; resistance decreased nonlinearly as conductive pathways formed between IDE fingers. 2 µL increments to 40 µL total; mean of n = 3 independently fabricated sensors, three replicates each; error bars = standard deviation. Calibration differences among formulations reflect variations in solution conductivity, surfactant concentration, and wetting on the porous LIG surface.

### 2.4 Sensor Calibration Drift Study

Repeated pesticide exposure produced a gradual, formulation‐dependent calibration drift that a simple water rinse could partially reverse, indicating that the LIG IDE can be refreshed and reused rather than discarded after each measurement. To quantify sensor recovery after rinsing, the post‐rinse calibration response was compared to the original Day 1 calibration. Percent recovery was calculated using:

$$\%\;Recovery=\frac{R_{Day\;7}-R_{Day\;6}}{R_{Day\;1}-R_{Day\;6}}\;*100$$

where R_Day 1_ is the original calibration resistance, R_Day 6_ is the resistance after six repeated pesticide exposure and drying cycles, and R_Day 7_ is the resistance after the tap water wash. A recovery value of 100% indicates that the post‐rinse response returned to the original Day 1 calibration. A calibration recovery of 0% indicates that the post‐rinse response remained equal to the Day 6 response, whereas 100% indicates complete restoration to the original Day 1 calibration. Values between 0 and 100% indicate partial restoration. Values greater than 100% indicate over‐recovery, where the response shifted beyond the original Day 1 calibration rather than returning to it exactly.

Using this approach, water rinsing produced formulation‐dependent recovery of the LIG IDE response, with positive recovery values ranging from 6% to 50% across the tested pesticide formulations. Specifically, the pesticide solution recovery values were 31.33 ± 15.66% for glyphosate (USDA), 6.75 ± 4.28% for S‐metolachlor, ‐42.97 ± 14.16% for glyphosate (retail), 10.16 ± 3.44% for propiconazole, 50.93 ± 29.83% for prothioconazole, and ‐111.88 ± 85.31% for gamma‐cyhalothrin. The greatest positive recovery was observed for prothioconazole, followed by glyphosate (USDA), while S‐metolachlor and propiconazole exhibited relatively limited restoration. In contrast, the negative recovery values obtained for glyphosate (retail) and gamma‐cyhalothrin indicate that rinsing shifted their responses farther from the original Day 1 calibration. These results demonstrate that the effectiveness of water rinsing was strongly formulation‐dependent and did not consistently restore the original sensor calibration. Day 7 and Day 8 calibrations approached the Day 1 response for several pesticide formulations, indicating that rinsing removed a portion of the residual pesticide deposits and partially regenerated the LIG sensing surface. However, complete restoration was not consistently achieved. The observed formulation‐dependent behavior may have resulted from differences in residual ionic content, surfactant composition, molecular adsorption, penetration of dried residues into the porous LIG network, and changes in surface wetting and liquid‐bridging behavior following rinsing [74, 75, 76]. Therefore, rinsing alone does not establish universal sensor reusability, and formulation‐specific cleaning protocols and recalibration would be required before reuse.

The pesticides listed in Table 2 were calibrated using sequential 6 µL droplets until a total deposited volume greater than 100 µL was achieved. The calibration procedure followed the same protocol as previously described. Following the initial calibration, the sensor was allowed to dry overnight under ambient conditions, and the calibration procedure was repeated the following day. This process was performed for six consecutive days to assess cumulative calibration drift caused by repeated pesticide exposure and drying. On days 7 and 8, the IDE sensors were rinsed with tap and deionized (DI) water, respectively, to evaluate whether residual pesticide deposits could be removed from the LIG surface and if the sensing response could be restored toward the original Day 1 calibration. As shown in Figure 5, the calibration curves progressively shifted from the original Day 1 response after each consecutive exposure cycle. Although the resistance values at higher accumulated volumes generally converged toward similar values across days, the largest deviations occurred during the initial droplet additions. This indicates that the low‐volume response of the sensor is most sensitive to residual surface contamination and changes in initial wetting behavior. The observed calibration drift is likely due to adsorption of both water and pesticide molecules onto the LIG. LIG and other graphitic nanomaterials have unique physicochemical properties such as high surface areas that contain large porous structures and defect sites that molecules can attach to [75, 77]. Therefore, LIG acts as the adsorbent surface, while water molecules, pesticide active ingredients, surfactants, and other formulation components act as adsorbates.

**FIGURE 5:**
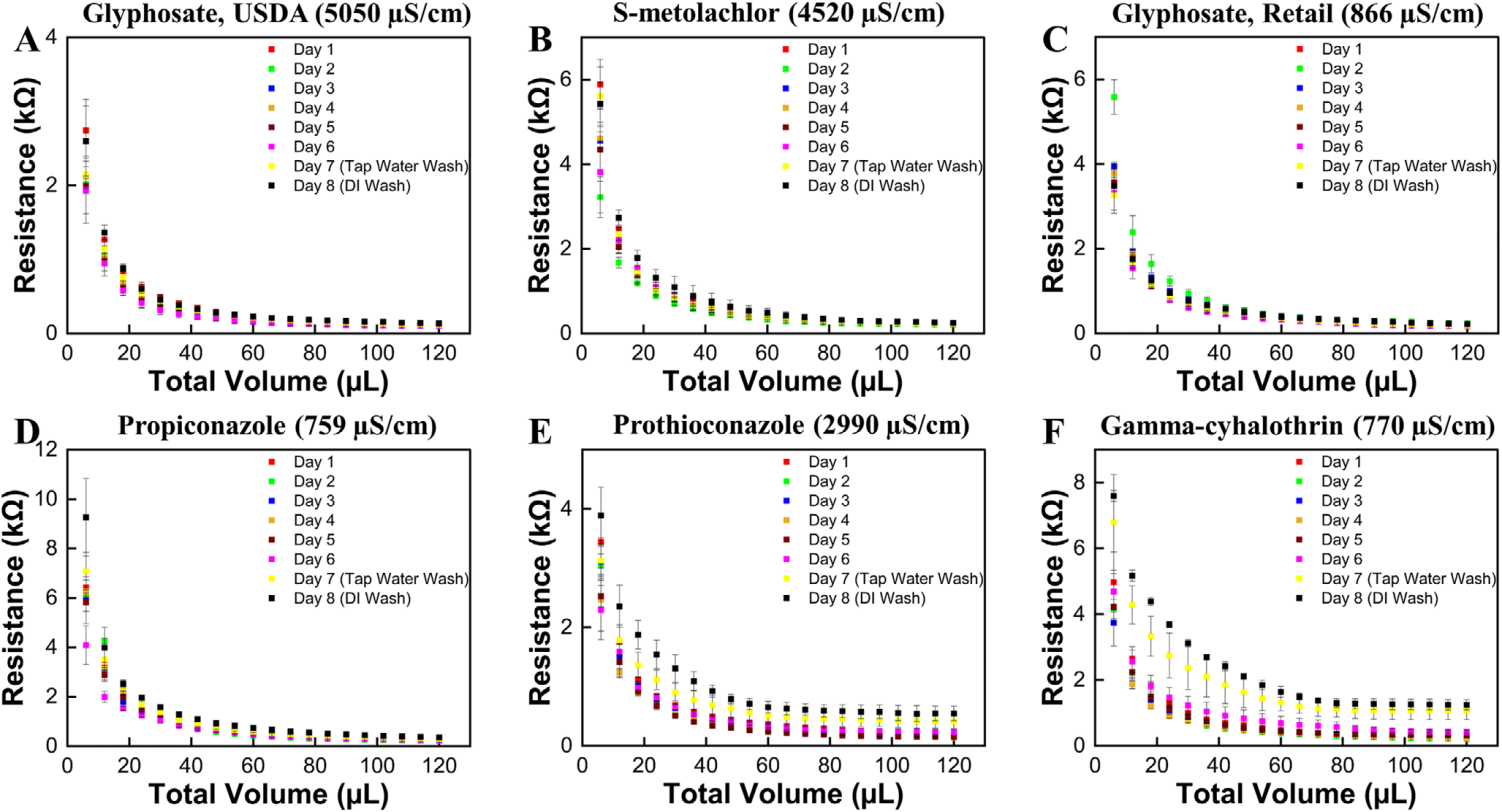
Calibration drift and recovery of the LIG IDE LWS over repeated daily exposure to spray formulations. Panels (A–F): (A) glyphosate, USDA (5050 µS/cm), (B) S‐metolachlor (4520 µS/cm), (C) glyphosate, retail (866 µS/cm), (D) propiconazole (759 µS/cm), (E) prothioconazole (2990 µS/cm), (F) gamma‐cyhalothrin (770 µS/cm). Resistance (kΩ, *y*‐axis) vs. cumulative volume (µL, *x*‐axis) from sequential 6 µL additions per daily cycle. Day 1, baseline of a freshly fabricated sensor; Days 2–6, repeated daily calibrations after overnight ambient drying. Day 7, 1 min tap water rinse + 24 h drying; Day 8, deionized water rinse under identical conditions. Recovery = (Wash Response − Day 6 Response)/(Day 1 Response − Day 6 Response) × 100 at matched volumes. Mean of n = 3 sensors, three replicates each; error bars = standard deviation. Drift increased over cycles, greatest at low volumes where sensitivity to residual adsorption is highest. Aqueous rinsing gave partial recovery toward Day 1 for all formulations, but incomplete recovery indicates some species remained adsorbed or absorbed in the porous LIG. Sensors are thus potentially reusable across multiple deployments, though periodic recalibration or replacement may be needed depending on formulation, exposure, and acceptable error.

The drift behavior observed in Figure 5 is believed to be governed primarily by adsorption onto the LIG surface rather than bulk absorption throughout the entire material. If the interacting molecules were predominantly retained near the surface of the porous LIG network, then rinsing with water would be expected to remove a substantial fraction of the deposited residues and partially recover the original resistance response. However, complete recovery was not consistently achieved, indicating that residual pesticide components remained on or within the LIG microstructure after repeated exposure and rinsing. This incomplete or formulation‐dependent recovery may result from a combination of strong molecular adsorption and partial penetration of dried pesticide residues into the porous LIG structure. As the pesticides dry on the surface of the LIG, the chemical molecules of these solutions have additional time to penetrate into the porous structure of the material, making it more difficult to completely remove any residue from the water wash, leading to contamination of the sensor [38, 78]. As a result, residual contamination can persist within the LIG microstructure, altering subsequent droplet spreading, electrode bridging, and measured resistance values. Overall, these results demonstrate that water rinsing can partially restore sensor performance after repeated pesticide exposure, but complete return to the original Day 1 calibration was not consistently achieved.

### 2.5 Environmental Impact Study

Environmental exposure had a substantially greater effect on sensor calibration than elevated temperature alone, with the largest calibration drift occurring for sensors exposed to combined heat, humidity, and precipitation (Figure 6). Among the tested pesticide solutions, the one containing prothioconazole exhibited the greatest sensitivity to environmental exposure, with the calibration coefficient (A) increasing from 16.52 ± 1.51 under control conditions to 53.99 ± 2.04 following outdoor deployment, corresponding to a 3.27‐fold increase. In comparison, the coefficient increased only to 22.01 ± 16.68 under greenhouse conditions, corresponding to a 1.33‐fold increase. These results indicate that prolonged exposure to humidity and precipitation contributed substantially more to calibration drift than elevated temperature alone.

**FIGURE 6:**
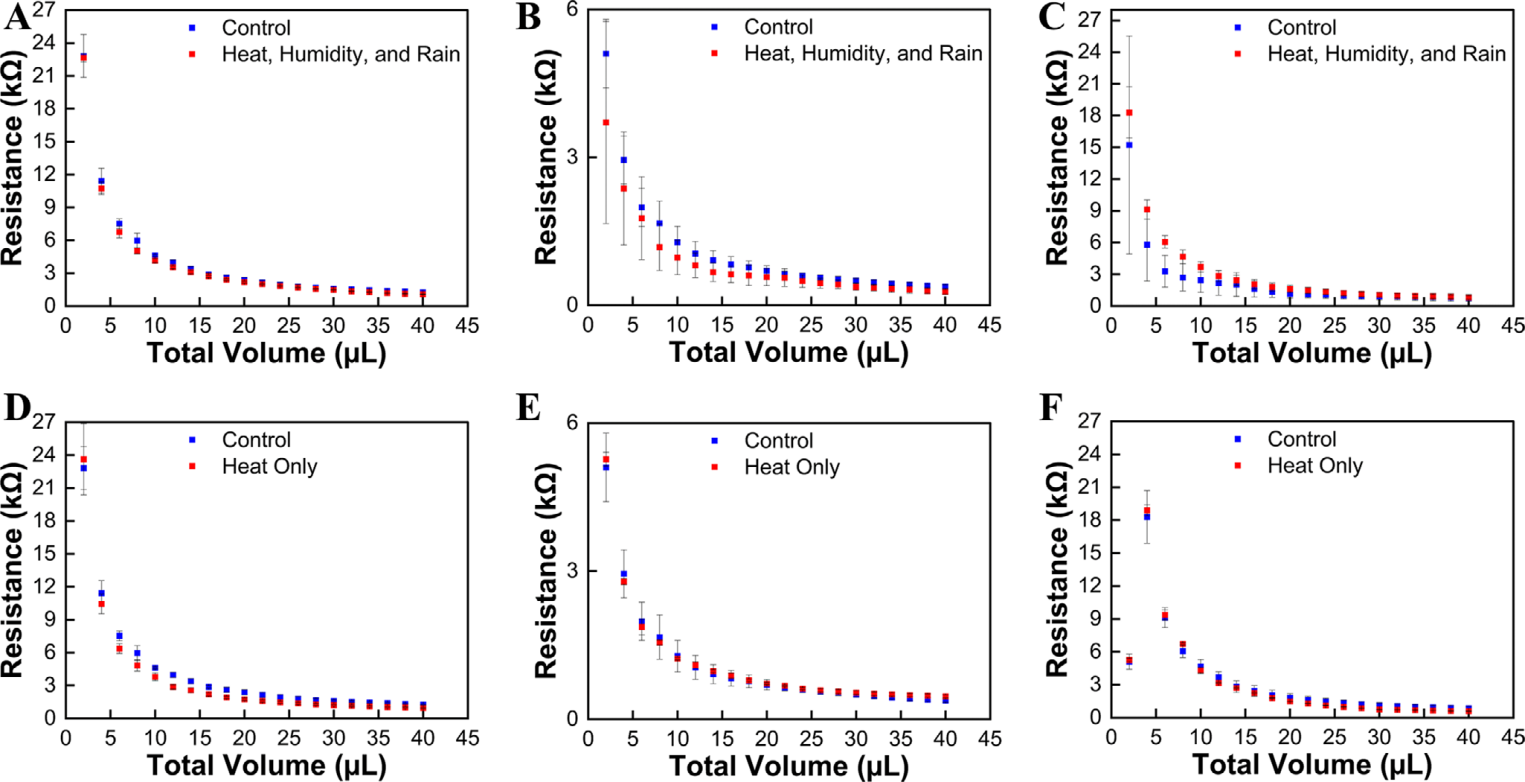
LIG LWS calibration response under different environmental exposures. Panels (A–C), control (lab) vs. combined heat, humidity, and outdoor rain; panels (D–F), before vs. after greenhouse heat‐only exposure. Solutions: (A & D) tap water, (B & E) glyphosate, USDA (5050 µS/cm), (C & F) prothioconazole (2990 µS/cm). Resistance (kΩ, *y*‐axis) vs. cumulative volume (µL, *x*‐axis) from sequential additions. Mean of n = 3 sensors, three replicates each; error bars = standard deviation. Three‐day deployments: outdoor (avg 80°F, max 90°F, 98% RH, 3.3 in. rain) or greenhouse (avg 85°F, 65% RH, no rain). Nonlinear calibration trends were retained after exposure, indicating the porous LIG network maintained function under thermal and environmental stress; small resistance shifts after heat/humidity/rain reflect changes in surface adsorption, residual moisture, and possible microstructural modification. Minimal drift supports reusable field deployment by reducing recalibration needs and improving reliability of pesticide deposition measurements under realistic field conditions.

To evaluate the effect of environmental conditions on sensor performance, three IDE sensors were deployed outdoors, and three additional sensors were placed in a greenhouse for three days. During this period, outdoor sensors experienced an average temperature of 80°F (maximum 90°F), average relative humidity of 98%, and 3.3 inches of precipitation, whereas greenhouse sensors were exposed to an average temperature of 85°F, 65% relative humidity, and no precipitation. Following exposure, the sensors were recalibrated using 2 µL droplets of tap water as well as 2 µL droplets for glyphosate (USDA)‐based and prothioconazole‐based pesticide solutions. These solutions were selected because they represented the high‐, intermediate‐, and low‐conductivity ranges of the pesticide formulations evaluated in this study.

As shown in Figure 6A,D, tap water calibrations were only minimally affected by environmental exposure. The calibration coefficient (A) decreased from 45.17 ± 1.84 under control conditions to 42.34 ± 2.73 following outdoor exposure (6.3% decrease) and to 44.47 ± 3.05 following greenhouse exposure (1.5% decrease). Similarly, the exponent (n) changed only slightly, from ‐0.957 ± 0.021 to ‐0.992 ± 0.011 outdoors and ‐1.060 ± 0.070 in the greenhouse, indicating relatively stable calibration behavior for tap water. The glyphosate (USDA) solution exhibited moderate calibration drift following environmental exposure. The coefficient (A) decreased from 9.59 ± 2.62 under control conditions to 7.94 ± 5.71 after outdoor deployment (17.2% decrease) and to 8.57 ± 0.70 after greenhouse deployment (10.6% decrease). The exponent (n) also shifted from ‐0.867 ± 0.051 to ‐0.794 ± 0.297 outdoors and ‐0.804 ± 0.038 in the greenhouse. Although these changes indicate some calibration drift, the magnitude of the drift was substantially smaller than that observed for prothioconazole‐based pesticide solution. Moreover, the largest environmental effects were observed for prothioconazole solution (Figure 6C,F). Following outdoor exposure, the calibration coefficient (A) increased from 16.52 ± 1.51 to 53.99 ± 2.04, corresponding to a 227% increase relative to the control calibration. In contrast, greenhouse exposure increased the coefficient to only 22.01 ± 16.68, corresponding to a 33% increase. The exponent (n) also shifted considerably under outdoor conditions, changing from ‐0.987 ± 0.065 to ‐1.247 ± 0.019, whereas greenhouse exposure resulted in a smaller change to ‐0.822 ± 0.318. These results demonstrate that calibration drift was amplified under combined heat, humidity, and precipitation conditions and was noticeable for lower‐conductivity pesticide formulations.

The greater calibration drift observed under outdoor conditions is likely associated with adsorption and partial absorption of water molecules within the porous LIG network. Previous studies have shown that graphitic materials containing high surface areas, interconnected pore structures, and defect‐rich regions readily interact with atmospheric moisture, particularly under prolonged exposure to elevated humidity and precipitation [63, 79]. These adsorbed molecules can alter the electrical conductivity and wetting behavior of the LIG surface, resulting in shifts in the resistance–volume calibration relationship. The relatively small drift observed under greenhouse conditions suggests that elevated temperature alone has a lesser impact on calibration stability than prolonged exposure to moisture. Future work should investigate surface modification strategies or protective coatings to reduce water adsorption and contaminant retention within the porous LIG structure while maintaining sensor sensitivity.

Sensor performance was evaluated in terms of between‐device reproducibility, repeated‐exposure stability, and short‐term environmental stability. Across three independently fabricated double‐lased 0.7 mm IDEs, the tap‐water calibration parameters corresponded to coefficients of variation of approximately 4.1% and 2.2%, respectively. These results indicate relatively low between‐device variation under the tested laboratory conditions. However, calibration stability depended strongly on the deposited formulation and exposure conditions. Repeated pesticide exposure over six daily calibration and drying cycles produced formulation‐dependent drift, particularly at low accumulated volumes. Three‐day greenhouse and outdoor exposures caused only limited changes in the tap‐water calibration but substantially greater drift for the prothioconazole‐based formulation under humid and rainy outdoor conditions. Therefore, the results support reproducible fabrication and short‐term operation under selected conditions but do not establish long‐term field reliability, shelf‐life stability, or universal calibration stability across pesticide formulations.

### 2.6 Spray Volume Validation

The LIG IDE calibration model accurately estimated spray‐deposited water volume at longer spray durations, with percent agreement values of 99.59% and 100.17% for 2 and 5 s spray times, respectively. The 1 s spray duration showed lower agreement at 65.65%, likely because the smaller deposited volume increased the influence of droplet variability, scale resolution, and nonuniform spray distribution across the IDE surface. For this experiment, three IDEs were placed onto the ground and sprayed with tap water using a handheld pesticide sprayer. Six feet was selected for the distance from the sprayer to the IDEs as this gave optimal distance for droplets to reach the sensor while being far enough away to not oversaturate the sensors with the water. Figure 7A illustrates the setup and distance for the droplets to travel to the IDEs. Before placing the IDEs on the ground, the sensors were weighed using a benchtop scale and then placed onto the ground to be tested. The three IDEs were sprayed with tap water for 1, 2, and 5 s. After the spraying was completed, the sensors were then weighed again. The change in weight was then divided by the density of water (1 mg/µL) to give a volume measured onto the surface of the sensor. Then each sensor was connected to the benchtop LCR where the resistance was measured. The measured resistance was inputted into Equation 1 using the coefficient (A) and exponent (n) for tap water in Table 2. Using this approach, the model produced accuracies of 65.65%, 99.59%, and 100.17% for 1, 2, and 5 s spray durations, respectively, as shown in Figure 7B. The close agreement at 2 and 5 s indicates that the LIG IDE sensor can quantitatively estimate deposited spray volume using the resistance‐based calibration model. The reduced agreement at 1 s suggests that very low spray deposition volumes may require additional replicate measurements or improved spray uniformity to reduce variability.

**FIGURE 7:**
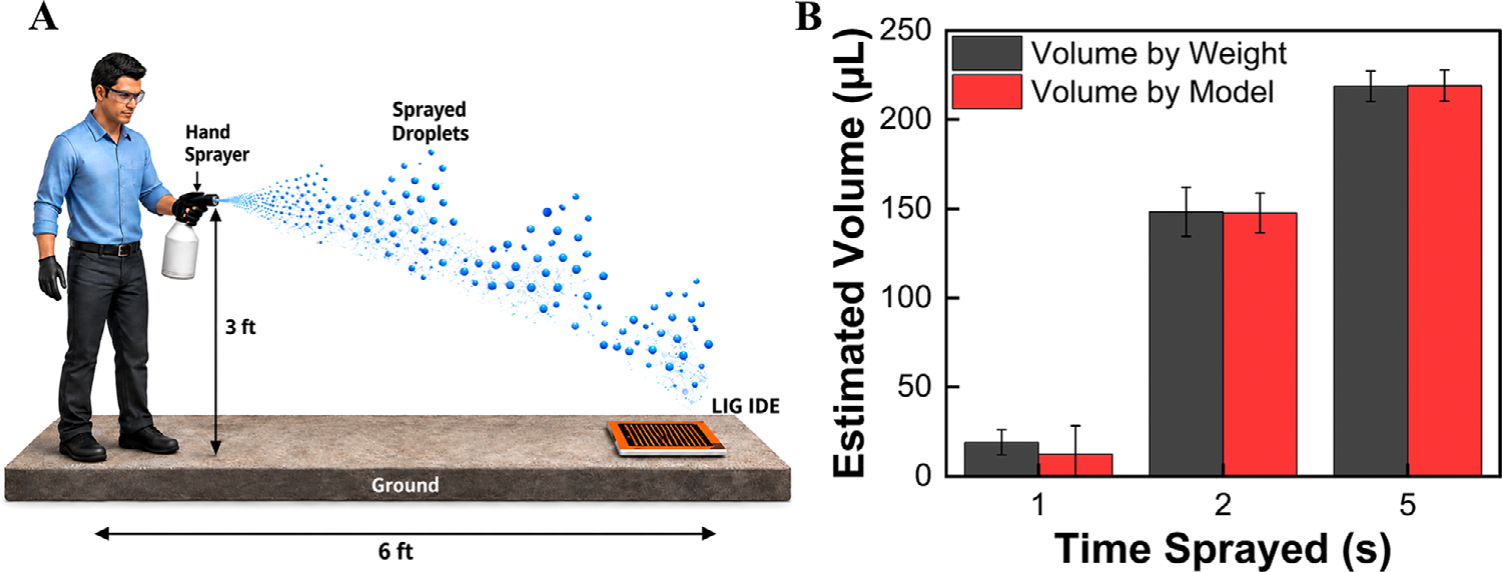
Spray volume validation of the LIG IDE LWS. (A) Schematic of the spray setup, with three IDEs placed on the ground 6 ft from a handheld sprayer and sprayed with tap water for 1, 2, and 5 s. (B) Deposited spray volume estimated by the resistance‐based calibration model (volume‐by‐calibration, Equation 1 with tap water A and n from Table 2) compared against the gravimetric reference (volume‐by‐weight), giving percent agreement of 65.65%, 99.59%, and 100.17% at 1, 2, and 5 s, respectively.

Next, the IDEs were compared against leaf wetness sensors from Onset. Three sensors were placed at the same locations as the IDEs and sprayed for 1, 2, and 5 s. The sensors were connected to a data logger that transmitted the total wetness of the sensor to an online server. To calculate the percent wetness of the LIG IDEs, the calculated volume of this experiment was divided by the maximum volume that could fit on the surface of the IDE. This maximum volume was determined by submerging the IDEs in a beaker of tap water and inputting the resistance value into Equation 1. It was determined that both the Onset LWS and LIG LWS exhibited similar percent wetness as shown in Table 3. Since both sensors have different geometries, the percent wetness was also calculated for the surface areas of the Onset LWS (23.97 cm^2^) and LIG LWS (20.10 cm^2^). When normalized, the Onset LWS gave slightly better percent wetness per surface area for the 1 s spraying time. For the 2 and 5 s spraying times, the LIG LWS gave a higher percent wetness per surface area, indicating improved performance compared to a commercially available LWS.

**TABLE 3:** Percent wetness comparison of Onset LWS and LIG LWS before and after normalization to surface area.

Time Sprayed (s)	Onset Wetness Sensor (%)	Onset Normalized Wetness (% cm^−2^)	LIG Wetness Sensor (%)	LIG Normalized Wetness (% cm^−2^)
1	4.30 ± 1.46	0.18 ± 0.06	3.04 ± 3.84	0.15 ± 0.19
2	30.15 ± 3.63	1.26 ± 0.15	36.05 ± 2.72	1.79 ± 0.14
5	45.75 ± 6.23	1.91 ± 0.26	53.45 ± 2.15	2.66 ± 0.11

The principal sustainability contribution of the proposed sensing approach is the potential to provide spatial feedback on pesticide deposition throughout a crop canopy. Identifying canopy regions receiving insufficient or excessive spray could allow subsequent nozzle settings, application rates, flight paths, or ground‐sprayer operation to be adjusted to improve coverage uniformity. Such feedback could reduce unnecessary pesticide application and limit off‐target movement into surrounding soil and water while maintaining adequate crop protection. However, pesticide‐use reductions, environmental loading, and agronomic outcomes were not directly quantified in this study. Practical deployment must also consider collection and disposal of polyimide‐ and silver‐containing sensors after contact with pesticide formulations. Future field studies should compare sensor‐guided and conventional spraying to quantify pesticide savings, coverage uniformity, runoff or drift reduction, and the environmental implications of single‐use and reusable deployment strategies.

## 3 Conclusions

In conclusion, this work reports the fabrication of a low‐cost, scalable resistive LIG LWS for monitoring pesticide spray distribution. The results demonstrate how changes in CO_2_ laser‐processing parameters tune the morphology and wettability of LIG. Across three independently fabricated sensors, the selected double‐lased LIG LWS exhibited a tap‐water power‐decay calibration with a coefficient (A) of 45.167 ± 1.841, an exponent (n) of ‐0.957 ± 0.021, and an R^2^ of 0.9921, while the calibration parameters varied among pesticide formulations. The LIG LWS demonstrated high accuracy as more droplets interact with the surface, up to over 100% compared to calculating the volume based on droplet weight. Additionally, the sensors showed promise when compared to commercial LWS from Onset, as the sensors calculated up to 8% more wetness when sprayed. When normalized to the surface area, this equated to an increase of up to 0.75% cm^−2^ compared to that of the Onset LWS. The LIG LWS has potential for in‐field operation during future work. However, repeated exposure and rinsing experiments demonstrated that the post‐rinse response was highly dependent on pesticide formulation. Water rinsing partially restored the original calibration for some formulations but produced little or negative recovery for others. Consequently, the present sensor should not be considered universally reusable. For applications requiring a low measurement error, a single‐use deployment strategy may be preferable. Reuse may be practical for selected formulations only after formulation‐specific testing, cleaning‐protocol optimization, and recalibration. The appropriate strategy should therefore be selected according to pesticide composition, exposure history, and the acceptable measurement uncertainty. Therefore, the current LIG LWS would need to be operated right after pesticide application to prevent further contamination of the sensors from environmental factors and further degradation of the LIG from the pesticides themselves.

Sensor stability was dependent on both the tested formulation and environmental exposure. Following three‐day exposure, the tap‐water calibration coefficient changed by only 1.5% under greenhouse conditions and 6.3% outdoors, whereas the prothioconazole‐based formulation exhibited changes of 33% and 227%, respectively. Repeated pesticide exposure also produced formulation‐dependent calibration drift, and the corrected rinsing results showed that restoration toward the original calibration was not consistent across all formulations. Therefore, the current sensor should not be considered universally reusable or calibration‐stable without formulation‐specific validation and recalibration. Nevertheless, the direct LIG fabrication process provides advantages including flexible electrode design, elimination of graphene‐ink preparation and post‐print annealing, and a simple two‐terminal resistance output suitable for distributed crop‐canopy sensing. Spatial measurements of pesticide deposition could support adjustments to spray operation that reduce overapplication and off‐target contamination while maintaining adequate crop coverage. Future work should evaluate long‐term aging, mechanical durability, LIG thickness and dimensional tolerances, protective surface treatments, formulation‐specific cleaning or disposable‐use strategies, portable multiplexed readout electronics, and field‐scale pesticide savings.

Hence, this manuscript presents an approach for utilizing the parameters of a CO_2_ laser to create a LIG LWS to quantify droplet spray deposition of various ready‐to‐spray pesticides. This laser allows for the creation of LIG in ambient conditions, and no additional materials are needed to waterproof the sensor during testing. This user‐friendly technology allows agricultural workers and professionals to monitor and map pesticide spray based on how wet their crop leaves are throughout an entire crop canopy. Additionally, this allows for a reduction in overapplication of pesticides while maximizing crop protection. Beyond pesticide spray monitoring, the developed LIG LWS could be utilized for precision irrigation management [80], early disease‐risk detection [52], and continuous microclimate monitoring in agricultural systems. Its lightweight, flexible, and low‐cost design also makes it suitable for integration into wearable plant sensors and environmental monitoring networks for smart agriculture applications [81]. Hence, we believe the LIG LWS, fabricated in a single‐lase step without the dielectric coating required by capacitive devices, can serve as foundational technology in precision agriculture and various environmental monitoring applications.

## 4 Experimental Section

### 4.1 Materials and Reagents

Kapton polyimide sheets (0.125 mm thick) were purchased from McMaster‐Carr. Silver (Ag) conductive ink (Sigma‐Aldrich, catalog no. 901769) was used to fabricate the electrical contact pads of the LIG sensor. The paste is a flexible, screen‐printable silver formulation designed for conductive tracks on polyimide substrates and contains a solid content of 44–46 wt.% at 130°C. The formulation contains conductive silver particles dispersed within a polymeric binder system that promotes adhesion to flexible substrates and maintains electrical conductivity after repeated mechanical deformation. The manufacturer did not report a particle size distribution for the silver particles in the product specifications. All pesticide solutions were commercial ready‐to‐spray formulations, in which the named active ingredient is one component alongside water, surfactants, and other proprietary adjuvants. For clarity, each solution is referred to throughout by its active ingredient, although the formulated product's surfactant and adjuvant content also contribute to its conductivity and wetting behavior. Two glyphosate‐based herbicides were evaluated: one obtained from collaborators through the USDA (5050 µS/cm) and one purchased retail from a local retail farm supplier (866 µS/cm), distinguished throughout as glyphosate (USDA) and glyphosate (retail). An S‐metolachlor‐based herbicide and a propiconazole‐based fungicide were also obtained from USDA collaborators, while a prothioconazole‐based fungicide and a gamma‐cyhalothrin‐based insecticide were purchased from a local retail farm supplier. A commercial LWS was purchased from Onset.

### 4.2 Fabrication and Characterization of LIG IDE

The LIG IDE was created on a Kapton polyimide sheet that was taped to an aluminum plate, washed with isopropyl alcohol, and placed on the bed of a 75 W CO_2_ Epilog Fusion M2 laser. The laser defocus was set to 1 mm with settings of 15% speed, 7% power, 50% frequency, and 1200 dots per inch (DPI). The design of the IDE was created in CorelDRAW. The laser converted the polyimide sheet into the LIG IDE. Once the first pass of the laser was completed, a second pass of the IDE design was performed by changing the laser speed to 20% and the laser power to 3%. A stencil was then placed on top of the polyimide sheet, and the laser was switched from raster to vector cutting. Vector cut settings were set at 15% speed and 3% power. The outline of the IDE legs was cut on the stencil, and tweezers were used to peel off the cut stencil to expose the IDE legs. Ag ink was painted onto the exposed IDE legs to protect the LIG and enhance the sensor conductivity. The rest of the stencil was peeled away to complete the IDE fabrication. The IDE was left to air dry in ambient laboratory conditions for 24 h to allow the Ag ink to completely dry.

### 4.3 Droplet Calibration Experiments

A 4263B LCR meter was used for all droplet and spray measurements. The LCR setup was a 2‐probe configuration with frequency set at 1 kHz and test voltage at 1000 mV. The IDE was connected to the LCR and measured the resistance with no tap water for 5 min to establish a baseline. Tap water droplets of 2, 4, 6, 8, 10, and 20 µL were pipetted onto the surface of the IDE. Once placed, a resistance value was measured after 10 s after placing a droplet onto the IDE. A total of 20 droplets of a single pipette volume were placed down the center of the IDE, and a resistance measurement was taken after each droplet. Once the last resistance value was measured, the IDE was washed with DI water, dried with air, and connected back to the LCR to test with the next pipette volume in sequence. After completing the experiment with tap water droplets, the same process was performed with the pesticides in Table 2 using 2 µL droplets.

### 4.4 LIG Drift Study

The LIG IDEs were tested to determine how each pesticide affects the calibration model after drying on the surface of the IDEs. Three IDEs were used for each pesticide, and 6 µL droplet calibrations were conducted for each pesticide. After the calibration was completed, the IDEs were left to air‐dry for 24 h in ambient laboratory conditions. After 24 h, the IDEs were calibrated again using the same droplet calibrations. This was done for 6 days, after which the IDEs were washed with tap water and dried for 24 h. After this 24‐h period, the droplet calibration was completed, and IDEs were washed with DI water. The IDEs were dried again for 24 h, and a final droplet calibration was performed to determine whether the original IDE calibration could be recovered by washing with tap or DI water.

### 4.5 Environmental Impact Study

Two sets of LIG IDEs were placed in two locations to see how varying environmental conditions affect the sensor performance. Three IDEs were placed outside and left for three days before collection and testing. The other three IDEs were placed inside a greenhouse and collected after three days. After collecting both sets of IDEs, they were immediately calibrated using 2 µL droplet calibrations previously described. During this period, outdoor sensors experienced an average temperature of 80°F (maximum 90°F), average relative humidity of 98%, and 3.3 inches of precipitation, whereas greenhouse sensors were exposed to an average temperature of 85°F, 65% relative humidity, and no precipitation.

### 4.6 Controlled Spray Experiments

The LIG IDEs were first weighed with a precision scale for dry weight. Then the IDEs were placed on concrete ground in the laboratory about 3 inches apart. A hand sprayer (Chapin 1‐Gallon) was placed 6 ft away and 3 ft above the position of the IDEs. The hand sprayer was pumped 10 times to build pressure inside the tank. The sprayer deposited mist tap water onto the surface of the IDEs for 1, 2, and 5 s. Once sprayed, the IDEs were connected to the LCR to obtain a resistance measurement. After the resistance measurement was obtained, the IDEs were weighed again for wetness weight. Both the resistance measurement and the change in IDE weight were used to calculate wetness percentages. The same experiment was performed with Onset LWS, and the resulting percent wetness after spraying was transmitted from a data logger to an online storage for analysis.

### 4.7 Data/Statistical Analysis

All experiments in the study were performed in a completely randomized design with n = 3 replicates. The results of the study were reported as mean ± standard deviation. Regression analysis of the droplet calibration experiments was performed by logging the droplet volume and corresponding resistance value and running a linear regression in R.

## Author Contributions

**Nathan M. Jared**: writing – original draft, writing – review and editing, investigation, methodology, formal analysis. **Kristi K. Bez**: validation, investigation. **Roham G. Goudarzi**: investigation, methodology, validation. **Griffin Ellis**: methodology, validation. **Krishangee Bez**: validation. **Emily A. Smith**: validation. **Jonathan C. Claussen**: conceptualization, investigation, funding acquisition, writing – review and editing, project administration, supervision.

## Conflicts of Interest

The authors declare no conflicts of interest.

## Supporting information




**Supporting File**: gch270165‐sup‐0001‐SuppMat.docx.

## Data Availability

The data that support the findings of this study are available from the corresponding author upon reasonable request.
