# Supplementary material for: Laser‐Induced Graphene‐Based Resistive Leaf Wetness Sensors for Pesticide Spray Deposition
Source: Glob Chall. 2026 Oct 15;10(10):e70165. doi: 10.1002/gch2.70165 (PMC13670206; doi:10.1002/gch2.70165)
Supplement: Supplementary file 1 [file GCH2-10-e70165-s001.docx]

**Supporting Information**

Laser-Induced Graphene-Based Resistive Leaf Wetness Sensors for Pesticide Spray Deposition

*Nathan M. Jared^1^, Kristi K. Bez^1^, Roham Goudarzi^1^, Griffin Ellis^1^, Krishangee Bez^2^, Emily A. Smith^2^ and Jonathan C. Claussen^1^**

^1^Department of Mechanical Engineering, Iowa State University, Ames, Iowa 50011, USA

^2^Department of Chemistry, Iowa State University, Ames, Iowa 50011, USA

^*^Author to whom correspondence should be addressed. Email: jcclauss@iastate.edu

**Table S1:** Raman spectra of LIG samples before and after applying the mask.

| **LIG Fabrication** | **D-Peak (cm^-1^)** | **G-Peak (cm^-1^)** | **2D-Peak (cm^-1^)** | **I_D_/I_G_** | **I_2D_/I_G_** |
| --- | --- | --- | --- | --- | --- |
| Hydrophilic without Mask | 1297.8 ± 0.1 | 1558.8 ± 0.2 | 2587.0 ± 3.0 | 2.30 ± 0.4 | 0.09 ± 0.05 |
| Hydrophilic with Mask | 1297.2 ± 0.2 | 1581.1 ± 0.3 | 2597.0 ± 3.0 | 2.22 ± 0.09 | 0.20 ± 0.07 |
| Hydrophobic without Mask | 1300.4 ± 0.2 | 1587.8 ± 0.3 | 2590.0 ± 1.0 | 2.30 ± 0.1 | 0.14 ± 0.03 |
| Hydrophobic with Mask | 1301.8 ± 0.1 | 1587.7 ± 0.2 | 2589.0 ± 2.0 | 2.4 ± 0.2 | 0.12 ± 0.03 |

**Figure S1:** X-ray Photoelectron Spectroscopy of (A) single-lased LIG before and after application of the mask and (B) double-lased LIG before and after application of the mask. Raman spectrum comparison of single- and double-lased LIG (C) before the application of the mask and (D) after the application of the mask.

**Table S2:** Calibration models for each LIG IDE design at varying droplet sizes.

|  | **SL 0.7mm Pitch** | | **SL 0.3mm Pitch** | | **DL 0.7mm Pitch** | |
| --- | --- | --- | --- | --- | --- | --- |
| *Droplet Size (µL)* | *Coefficient (A)* | *Exponent (n)* | *Coefficient (A)* | *Exponent (n)* | *Coefficient (A)* | *Exponent (n)* |
| 2 | 32.162 | −0.824 | 9.326 | −0.841 | 43.964 | −0.974 |
| 4 | 40.274 | −0.93 | 11.303 | −0.987 | 44.116 | −0.97 |
| 6 | 27.476 | −0.785 | 5.904 | −0.668 | 43.949 | −0.961 |
| 8 | 36.076 | −0.868 | 13.770 | −0.931 | 48.531 | −0.976 |
| 10 | 38.607 | −0.88 | 13.342 | −0.843 | 46.802 | −0.952 |
| 20 | 43.806 | −0.868 | 22.719 | −0.929 | 43.907 | −0.914 |
| **AVG** | 36.399 | −0.859 | 12.727 | −0.867 | 45.167 | −0.957 |
| **SD** | 5.363 | 0.045 | 5.187 | 0.103 | 1.841 | 0.021 |

**Figure S2:** Experimental setup of droplet calibration of LIG LWS using an LCR meter by depositing droplets of calibration solution with a pipette.

**Figure S3:** Calibration curve of 2 µL water droplets onto the LIG LWS with a 0.7 mm pitch and double-lased fabrication technique across n = 4 sensors.
